# Risk factors and long-term outcomes in anterior iliac and obturator hip dislocation

**DOI:** 10.1007/s00068-025-02885-9

**Published:** 2025-05-17

**Authors:** Vera Jaecker, Stephan Regenbogen, Sven Märdian, Hanno Brinkema, Ulrich Stöckle, Sven Shafizadeh

**Affiliations:** 1 Center for Musculoskeletal Surgery, Charitè – University Medicine Berlin, Augustenburger Platz 1, 13353 Berlin, Germany; 2 Department of Trauma and Orthopedic Surgery, Cologne Merheim Medical Center, Witten/Herdecke University, Ostmerheimer Str. 200, 51109 Cologne, Germany; 3 Berufsgenossenschaftliche Unfallklinik Murnau, Department of Traumatology and General Surgery, Professor-Küntscher-Str. 8, 82418 Murnau, Germany; 4 Berufsgenossenschaftliche Unfallklinik Ludwigshafen, Department of Traumatology, University of Heidelberg, Ludwig-Guttmann-Str. 13, 67071 Ludwigshafen Am Rhein, Germany; 5 Department of Trauma, Hand and Reconstructive Surgery, Rostock University Medical Center, Schillingallee 35, 18057 RostockRostock, Germany; 6 Department of Orthopedic Surgery and Sports Traumatology, Witten/Herdecke University, Sana Medical Centre, Aachener Str. 445-449, 50933 Cologne, Germany

**Keywords:** Traumatic anterior hip dislocation, Obturator hip dislocation, Avascular necrosis, Pipkin fracture, Femoroacetabular impingement (FAI)

## Abstract

**Purpose:**

Traumatic anterior hip dislocation is a severe but poorly studied injury. This study aimed to analyze characteristics, risk factors and prognostic factors regarding long-term morbidity and outcomes in patients who had sustained traumatic anterior hip dislocation.

**Methods:**

Demographics, injury mechanism, and treatment-related characteristics of patients with anterior hip dislocations at three level-one trauma centers from 2009–2023 were analyzed. Acetabular and femoral morphology were assessed using CT scans to identify anatomical risk factors. Incidence of avascular necrosis (AVN), post-traumatic osteoarthritis (PTOA), further complications, return to work and sports, and patient-reported outcomes (PROMs), including Tegner Activity Scale (TAS) and modified Harris Hip Score (mHHS) were recorded at the follow-up.

**Results:**

Out of 196 patients with traumatic hip dislocations, 19 anterior dislocations (12 iliac anterosuperior and 7 obturator) were identified. Ipsilateral knee injuries occurred in 36.8%, and 73.7% had concomitant femoral head or acetabular rim fractures. Obturator dislocations were commonly simple dislocations, while iliac dislocations involved more complex associated fractures often requiring surgery. Acetabular anteversion and cam-type femoroacetabular impingement (FAI) were identified as risk factors. Twelve patients (63%) were available for follow-up (mean 8.33 ± 5.05 years). The majority demonstrated good to excellent mHHS (mean 86.9), and minimal TAS decrease (5.33 to 4.67). AVN was not observed, and only one patient required hip arthroplasty following PTOA.

**Conclusion:**

Anterior hip dislocations commonly result from high-energy “dashboard" injuries, with acetabular anteversion and cam-type FAI morphology being contributing risk factors. Long-term functional outcomes were favorable, with low rates of avascular necrosis or osteoarthritis, independent of type and complexity of the dislocation.

## Introduction

Dislocation of the native hip represents a potentially severe injury that most commonly results from high-energy trauma. It is often associated with complications, including avascular necrosis (AVN) of the femoral head, post-traumatic osteoarthritis (PTOA), nerve injury, and long-term limitations in activities of daily living [1, 2]. While posterior dislocations have been studied more extensively, anterior dislocations remain poorly understood due to their rarity, representing only 8–10% of all hip dislocations [2–4]. It has been postulated that a ‘dashboard injury’, including an impact to the knee with the hip position in flexion and adduction, represents the critical factor causing posterior hip dislocation. It is similarly thought that anterior hip dislocations result from high-energy trauma with the hip in abduction and external rotation, with the potential for an anteroinferior obturator dislocation or an anterosuperior iliac dislocation. With a higher degree of flexion, the femoral head dislocates below the pubofemoral ligament, resulting in a dislocation of the femoral head to the obturator foramen [5, 6]. When the hip is in a more extended position, the femoral head dislocates anterosuperiorly between the iliofemoral and pubofemoral ligaments, resulting in an anterosuperior iliac dislocation that may be associated with an avulsion fracture of the anterior inferior iliac spine [5]. It is common for anterior hip dislocations to be accompanied by fractures of the femoral head or acetabular wall, with rates up to 78% reported in the literature [3, 4, 7, 8]. Nevertheless, the precise mechanism underlying the trauma mechanism remains uncertain. The reasons why some hips dislocate anteriorly while others dislocate posteriorly in response to identical accident mechanisms remain poorly understood.

In posterior hip dislocations, there is growing evidence that, in addition to the trauma mechanism, patients with specific risk factors, such as acetabular retroversion and cam-type femoroacetabular impingement (FAI) are more prone to these injury patterns, regardless of the intensity of the trauma [9–12]. However, potential risk factors contributing to anterior hip dislocations remain unknown. Further evidence surrounding characteristics, functional outcome, and prognostic factors of anterior hip dislocations is based on case reports and few series of patients with small sample sizes.

The objective of this study was to analyze the mechanism of trauma, type of dislocation, concomitant injuries, treatment, and complications for patients with traumatic anterior hip dislocations and to compare the characteristics between anterosuperior iliac and obturator dislocations.

Furthermore, the study aimed to assess patient-reported outcome measures (PROMs) over a 14-year period, with a minimum of two years of follow-up. It was hypothesized that specific prognostic factors (e.g., epidemiological variables, timing of hip reduction, associated injuries) could be identified as influencing the development of AVN or PTOA, PROMs, and return to sports.

Moreover, the study sought to identify acetabular and femoral risk factors for anterior hip dislocations by examining the hip morphology for FAI deformities, acetabular version and coverage in these patients and comparing the data to those of hips dislocated posteriorly and to normative data.

## Materials and methods

Following the approval of the institutional review board, all consecutive patients who had sustained a traumatic anterior dislocation of their native hip at three level one trauma centers between 2009 and 2023 were subjected to analysis. The study included patients aged over 18 years with closed growth plates at the time of injury who had sustained a traumatic anterior hip dislocation. Patients were excluded if their medical records lacked sufficient detail regarding the trauma mechanism or treatment, or if their computed tomography (CT) scans of the pelvis were unavailable.

Patient demographics (age, gender, body mass index), trauma mechanism, concomitant injuries, time to reduction, treatment, and complications were assessed. The analysis of radiological imaging included the direction of dislocation and the differentiation between anteroinferior obturator dislocation and anterosuperior iliac dislocation. Moreover, concomitant femoral head and acetabular fractures were documented and associated femoral head fractures classified according to the Pipkin classification system [13].

### Follow-up and patient-reported outcome measures (PROMs)

All patients who met the inclusion criteria were contacted by the responsible orthopedic surgeon from each of the three participating trauma centers and provided with a study information letter and written consent form. Once written consent was obtained, the patients were included in the follow-up investigation. Patients with cognitive disorders or paraplegia were excluded from the follow-up investigation.

Subsequent history was also documented, including instances of re-dislocation or hip instability, the emergence of AVN or PTOA, additional surgical procedures, residual nerve damage, and sexual dysfunction, including erectile dysfunction in men and loss of libido in women. The level of sports activity before and after the injury was documented, as well as the ability of patients to resume their previous level of participation in sports and return to work. Moreover, the activity level prior to and following the injury was determined using the Tegner Activity Scale (TAS) [14]. Activities of daily living, functional outcome and pain were assessed using the validated modified Harris Hip Score (mHHS) [15, 16]. The results of mHHS were classified as follows: < 70 poor, 70–79 moderate, 80–89 good, and > 90 excellent.

### Measurements of the acetabular and femoral morphology

CT scans were recalculated and reformatted for further analysis into true axial and coronal planes and analyzed using a bone window with a slice thickness of 1 mm.

The following measurements were used to analyze acetabular FAI morphology, acetabular coverage, and dysplasia based on standardized and validated measurement techniques as previously described [9]. In the coronal plane, the acetabular index, the lateral center–edge angle and the acetabular depth/width ratio were quantified. In the axial plane, the cranial acetabular anteversion angle, the central acetabular version angle, and the anterior (AASA) and posterior acetabular sector angles (PASA) were measured.

The femoral head and neck concavity (cam-type FAI) was evaluated by measuring the alpha angle in the coronal and axial planes as previously described [9]. The assessment of coxa valga or vara was conducted by measuring the caput-column-diaphyseal (CCD) angle [17].

All measurements were conducted by two experienced orthopedic surgeons on both the injured and unaffected hips in each patient. The intraclass correlation coefficient (ICC) was calculated for the two observers’ measurements and indicated almost perfect agreement [18].

The obtained measurements were then compared to previously published data on posterior hip dislocations and normative data [9].

### Statistics

The statistical analysis was conducted using Python data science (Jupyter Notebook Software for Statistical Computing, version 3.8.16). Descriptive statistics, including means, frequency counts, percentages, and ranges were determined as appropriate for continuous and categorical variables. Data were tested of normality and homoscedasticity using the Shapiro–Wilk and Levene's test, respectively. Depending on these results, a parametric or non-parametric test (Mann–Whitney U. A Chi-square or Kruskal–Wallis test) was performed to detect differences between subgroups. Significance was set at *p* < 0.05.

## Results

### Patient characteristics, trauma mechanism and concomitant injuries (Tables 1 and 2)

**Table 1:** Demographics of patients with traumatic anterior hip dislocation

	All patients (*n* = 19)	
Age at time of injury (years)	45.47 ± 20.45	
Gender		
Male	13	
Female	6	
Side		
Right	8	
Left	11	
BMI	23.78 ± 4.18	
Injury mechanism		
Motorcycle accident	5	
Suicidal collision with train	3	
Motor vehicle accident	2	
Skiing	3	
Fall from height	4	
Minor trauma	2	
Duration of hospital stay (days)	20.36 ± 20.14	

The values are given as numbers or mean values ± SD (standard deviation)

*BMI* Body Mass Index

**Table 2:** Type of anterior hip dislocation and associated injuries

	All patients with anterior hip dislocations (*n* = 19)	Iliac/anterosuperior dislocation (*n* = 12)	Obturator dislocation (*n* = 7)
Associated fractures			
Femoral Head	9	5	4
- Pipkin I	3	0	3
- Pipkin II	1	1	0
- Pipkin III	0	0	0
- Pipkin IV	5	4	1
Isolated anterior acetabular wall	5	3	2
None (simple dislocation)	5	4	1
Associated injuries			
Mono trauma	2	2	0
Multiple injuries	3	2	1
Polytrauma (ISS ≥ 16)	14	8	6
Ipsilateral knee injury			
Isolated PCL injury	2	0	2
Knee dislocation	3	1	2
Patella fracture	2	1	1
None	12	10	2
Primary nerve injury			
Yes	2	1	1
No	17	11	6

The values are given as numbers

*ISS* Injury Severity Score

**Fig. 1:**
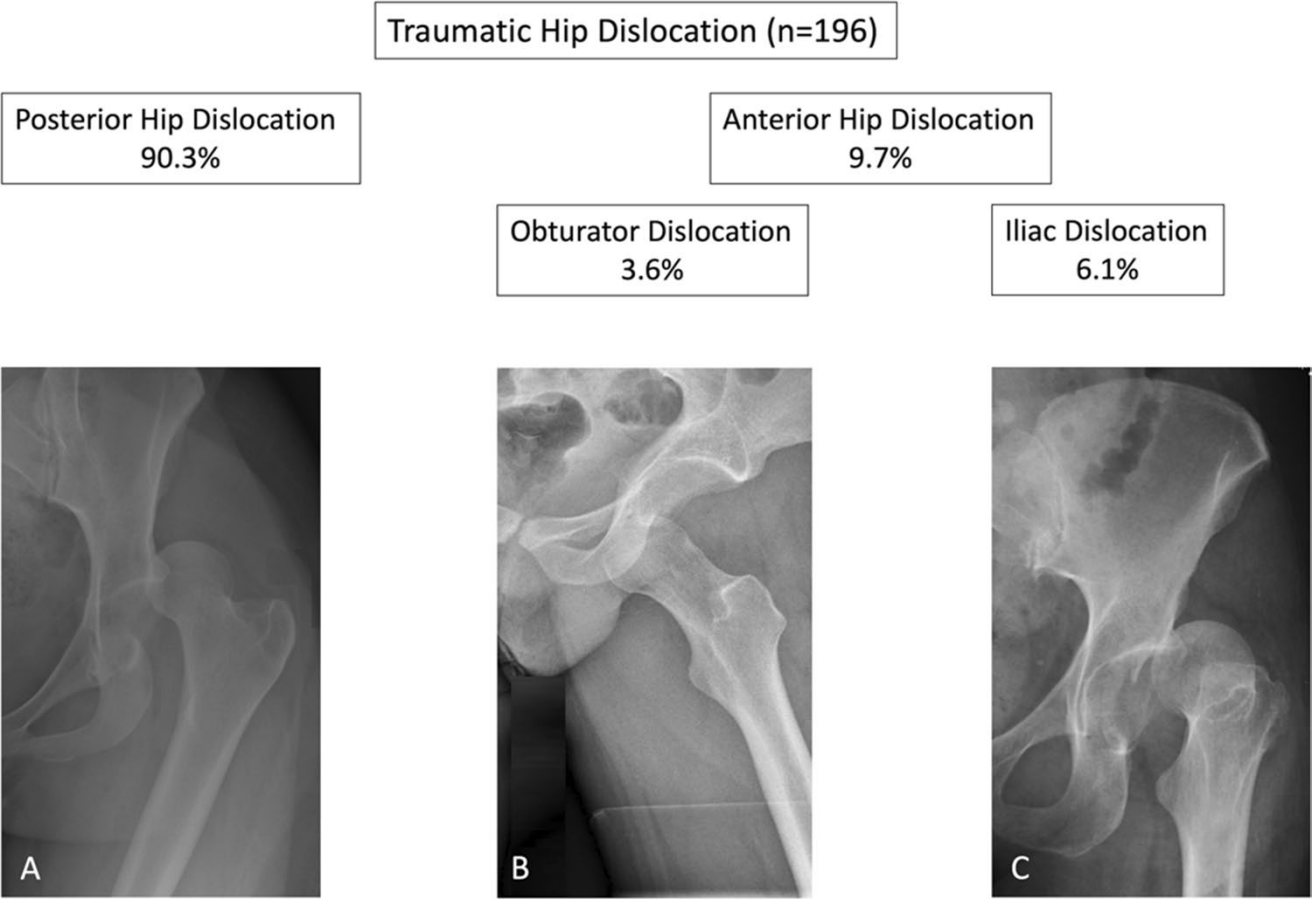
Distribution of traumatic hip dislocations. Posterior dislocations represent the majority (**A**). Anterior dislocations can be further subdivided into obturator dislocations (**B**) and anterosuperior iliac dislocations (**C**)

A total of 19 patients met the pre-established inclusion criteria and were subsequently subjected to analysis. This equates to 9.7% of the 196 cases of hip dislocation that were treated at our institutions over the 14-year period (Fig. 1). The mean age at the time of injury was 45.47 ± 20.45 years. A high-energy trauma mechanism was identified 17 patients (89.5%) and 14 patients (73.7%) sustained a polytrauma with an Injury Severity Score (ISS) of ≥ 16 points. Associated knee injuries were observed in 7 patients (36.8%) of the cases and predominantly occurred in patients who sustained an obturator dislocation.

#### *PCL* Posterior cruciate ligament

### Associated fractures of the femoral head or acetabulum (Table 2)

Computed tomography (CT) was available in all cases and used for the radiological analysis regarding the type of dislocation and associated fractures of the femoral head (Pipkin Type I-IV) or the acetabular rim. Twelve cases were observed to have iliac anterosuperior hip dislocation, and 7 cases exhibited obturator dislocation.

Associated fractures were observed in 14 cases (73.7%). Femoral head fractures and acetabular fractures were observed in 9 and 10 cases, respectively, while a combination of both (Pipkin type IV) was observed in 5 cases (Table 2). Isolated femoral head fractures were more frequently observed in cases of obturator dislocation, while Pipkin type IV fractures were more prevalent in patients with iliac dislocation (Table 2).

### Treatment (Table 3)

**Table 3:** Primary treatment of patients with anterior hip dislocations

	All patients (*n* = 19)	Iliac/anterosuperior dislocation (*n* = 12)	Obturator dislocation (*n* = 7)
Time to hip reduction			
< 6 h	15	8	7
6–24 h	2	2	0
> 24 h	2	2	0
Treatment			
Only closed reduction	11	5	6
Open reduction following unsuccessful closed reduction	1	1	0
Closed reduction followed by a two-stage surgery	6	5	1
Direct open reduction	1	1	0
Surgical technique			
Open capsulolabral refixation	4	4	0
ORIF	2	2	0
Arthroscopy*	2	1	1

The values are given as numbers ± standard deviation (SD)

ORIF = Open reduction and internal screw fixation of the pipkin fragment

* Arthroscopy with removal of intraarticular fragments

Hip reduction was predominantly performed within 6 h. In 2 cases, hip reduction was beyond 24 h. One case was an unsuccessful primary closed reduction, and one was a direct open reduction with screw fixation due to an intra-articular fragment.

Eleven patients (57.9%) were treated conservatively, solely through closed reduction.

A surgical treatment was mainly performed in iliac dislocations. The most common surgical procedure was an open capsulolabral refixation of the acetabular rim and/or open reduction and internal screw fixation of the Pipkin fragment (Table 3).

### Follow-up

One patient was excluded from the follow-up examination due to the presence of paraplegia, and three patients were excluded due to an inability to provide informed consent as a result of psychiatric illnesses. Of the remaining 15 eligible patients, 12 (80%) were available for follow-up. The mean time to follow-up was 8.33 ± 5.05 years (range, 3 to 15 years). The mean age of the subjects at the time of follow-up was 48.20 ± 15.55 years.

No cases of re-dislocation of the native hip were observed, and only one patient reported a residual subjective instability. One patient reported the persistence of impaired sensation following initial damage to the sensomotoric sciatic nerve.

A single patient developed PTOA and underwent a total hip arthroplasty (THA).

### Activity level and patient-reported outcome measures (PROMs) (Table 4)

**Table 4:** Clinical outcomes and patient-reported outcome measures (PROMs)

Time to follow-up (years)	8.33 ± 5.05 (3–15)
Age at follow-up	48.20 ± 15.55 (29–68)
BMI at follow-up	24.72 ± 7.07
Gender	
Male	9
Female	3
Modified Harris Hip Score	86.87 ± 5.13 (77–91)
Tegner Activity Scale	
Preinjury	5.33 ± 3.08 (2–10)
At follow-up	4.67 ± 2.94 (2–10)
Sexual disorders	
Yes	1
No	11
Return to Work	
Yes	10
No	0*
Limitations in sports activities (compared to preinjury)	
None	9
Mild	3
Moderate	0
Highly	0
Post-traumatic degenerations	
Osteoarthritis	1
Avascular necrosis	0
Subsequent surgery	
THA	1
Arthroscopy	1

Patients available for follow-up: *n* = 12

The values are given as numbers or mean values ± SD (standard deviation) and ranges in parentheses.

*THA* Total Hip Arthroplasty

*Two patients retired

The majority of patients (11 out of 12) demonstrated good or excellent results in the mHHS, with only one patient exhibiting a moderate result. The mean TAS was 5.33 (median 5.5) ± 3.08 prior to the injury, demonstrating a slight decline to 4.67 (median 4.5) ± 2.94 at the time of follow-up. However, all patients reported that they had no or only mild limitations in their pre-injury level of sport. Furthermore, all patients were able to return to their previous work. A comparison of the PROMs between iliac and obturator dislocations revealed no statistically significant differences (*p* > 0.05).

### Acetabular and femoral morphology (Table 5)

**Table 5:** CT measurements assessing acetabular morphology of posteriorly dislocated hips compared with controls

CT measurement	Anterior hip dislocations (*n* = 19)	Posterior hip dislocations† (*n* = 83)	Normative Data† (*n* = 83)
Lateral center–edge angle (°)	36.91 ± 7.26	36.24 ± 8.30	37.45 ± 8.08
Acetabular Index (°)	4.91 ± 6.90	4.65 ± 5.09	3.04 ± 4.45
Acetabular Depth/Width ratio	302.80 ± 59.80	318.20 ± 53.30	319.46 ± 44.10
Cranial acetabular version angle (°)	19.58 ± 9.39*	11.05 ± 7.45	13.13 ± 6.55
Central acetabular version angle (°)	21.93 ± 5.18*	14.72 ± 5.32	19.50 ± 5.29
Anterior acetabular sector angle (°)	56.29 ± 8.42*	63.32 ± 10.79	64.01 ± 9.29
Posterior acetabular sector angle (°)	103.50 ± 10.12	93.58 ± 10.41	102.07 ± 8.77
Alpha angle in the coronal plane (°)	57.60 ± 8.90*	55.72 ± 11.52	46.69 ± 7.63
Alpha angle in the axial plane (°)	38.16 ± 11.47*	41.48 ± 11.76	34.24 ± 6.34
CCD angle (°)	132.28 ± 4.85	133.55 ± 5.48	131.37 ± 5.67

The values are given as numbers ± standard deviation (SD); *p < 0.05, compared to normative data; †previously published data [7]

Computed tomography (CT) measurements of the femur and acetabulum were conducted on all 19 patients. A comparison between iliac and obturator dislocations revealed no statistically significant differences (*p* > 0.05). The data were compared with previously published data on posterior hip dislocations and a normative control group [9].

Patients with anterior hip dislocations were observed to exhibit a greater proclivity for acetabular anteversion, characterized by markedly increased cranial and central acetabular version angles and decreased AASA, in comparison to posterior dislocations and the control group (*p* < 0.001). Furthermore, the alpha angles in the coronal plane were found to be significantly higher in comparison to the control group, indicative of a radiological sign for a cam-type FAI (*p* < 0.001).

## Discussion

The main findings of this study were that although high-energy trauma was the most common mechanism of injury, traumatic anterior hip dislocations were associated with only minor limitations in activities of daily living, sports, and return to work at mid- to long-term follow-up. All patients had sustained high-energy trauma, with a dashboard injury being the most common mechanism, especially for obturator dislocations. In addition, acetabular anteversion and cam-type FAI morphology could be identified as contributing anatomical risk factors. Regardless of the occurrence of obturator or anterior iliac hip dislocation, patients were more likely to have acetabular anteversion compared to posterior dislocations and normative data [9]. Associated fractures of the femoral head or acetabulum were predominantly observed. Most of the obturator dislocations were associated with a simpler fracture pattern and were treated non-operatively. In contrast, iliac dislocations tended to have more complex associated fractures and were mainly treated operatively. However, the generally assumed risk of sustaining AVN or PTOA was low.

In light of the limited number of existing observational studies, the results of the presented study reinforce the hypothesis that high-energy trauma represents a crucial contribution to anterior hip dislocation in 90% of cases. While posterior hip dislocations are associated with lower-energy or sports-related mechanisms in up to 20% of cases [2], a minor trauma was observed in 10.5% of patients with anterior hip dislocations.

Similar to associated injuries in posterior hip dislocations, knee injuries, especially to the PCL, were also found in anterior hip dislocations in one third of the patients. Therefore, the question of why some hips dislocate anteriorly rather than posteriorly in ‘dashboard-type’ injury mechanisms is of interest. It can be discussed that in addition to the axial compression force responsible for posterior hip dislocations, an additional force vector, such as abduction or a rotational force, is involved in high-energy mechanisms leading to anterior hip dislocation. This may be particularly applicable in cases where an increased acetabular anteversion is present, as observed in the present study, whereas posterior hip dislocations are more commonly associated with acetabular retroversion [9, 10]. These predispositions may contribute to the femoral head being levered out of the acetabulum in an anterior direction. Since anatomical variations have been reported in posterior hip dislocations in several studies [9, 11, 19–21], and the understanding of the exact underlying mechanism in association with anatomical factors is unclear, biomechanical follow-up studies investigating these questions would be of considerable interest.

While previous literature reports rates of concomitant fractures in anterior hip dislocation ranging from 15 to 78% [3–5], a rate of 73.7% was observed in the presented study. However, other studies have employed a variable use of cross-sectional imaging, and the analysis of CT scans was conducted in all cases in this study, allowing for a more precise evaluation of accompanying fractures.

In accordance with previous findings of anterior hip dislocations by Wojahn et al. [4], most obturator dislocations were being associated with a simpler fracture pattern of the femoral head, while iliac dislocations tended to have more complex associated fractures, especially combined acetabular and femoral head Pipkin IV fractures. The study revealed additional differences between iliac and obturator dislocations. In the majority of obturator dislocations, non-operative treatment was feasible, whereas open reduction and capsulolabral refixation was the most common procedure for iliac dislocation. However, no differences were identified regarding the functional outcome between obturator and iliac dislocations. This finding contrasts with the results reported by Wojahn et al., who reported superior functional outcomes for obturator dislocations [4]. However, the sample sizes were underpowered to offer a definite conclusion on the different dislocation types and other predictive variables such as reduction time and surgical treatment.

The present study's data demonstrate good functional outcomes, which are superior to those observed in cases of posterior hip dislocations [2]. It can be hypothesized that fractures of the anterior acetabular wall have a less significant impact on hip biomechanics and the patient's functional capacity than posterior wall fractures in posterior dislocations. The average modified Harris Hip Score at a mean follow-up of 5.3 years was 86.9. Although femoral head fractures are generally considered as being associated with AVN, this correlation was not observed in the anterior hip dislocations with concomitant femoral head fractures examined in the present study. This may be due to the blood supply to the head of the femur through the posterior capsule, which may be compromised during posterior hip dislocation but may remain intact during anterior hip dislocation. Furthermore, only one elderly patient who had sustained an iliac dislocation with an anterior wall fracture was converted to a THA following PTOA.

Wojahn et al. also observed no AVN, and delayed conversion to THA was relatively low, with only one of 16 patients following anterior hip dislocation. Similarly, Bastian et al. observed only one out of ten cases with AVN [3].

This study has strengths and limitations. Due to the rarity of anterior hip dislocations, information on the trauma mechanisms, treatment, prognostic factors, and risk factors are based on case reports and few case series. This study includes one of the largest series of anterior hip dislocations and longest follow-up period in the current literature. Although it is difficult to draw conclusions regarding small subgroup analyses, the results are relevant in managing this injury because surgeons may need more experience to rely on. In addition, this study first analyzed morphological acetabular and femoral risk factors contributing to anterior hip dislocation. As most of the patients sustained polytrauma, associated injuries may have affected the functional outcome. However, this strengthens the results because most patients had good or excellent functional outcomes.

In conclusion, the most common cause of anterior hip dislocation was a high-energy"dashboard"injury. Additionally, acetabular anteversion and cam-type FAI morphology could be identified as contributing risk factors. In the majority of cases, concomitant femoral head or acetabular fractures were observed, with obturator dislocations being associated with a relatively simple fracture pattern, whereas iliac dislocations tended to have more complex concomitant fractures. However, independent of the direction of anterior hip dislocation, only minor limitations in activities of daily living, sports, and return to work were observed at mid- to long-term follow-up, with a low risk of post-traumatic femoral head necrosis or osteoarthritis.

## Data Availability

No datasets were generated or analysed during the current study.
